# Supplementary material for: BuDDI: Bulk Deconvolution with Domain Invariance to predict cell-type-specific perturbations from bulk
Source: bioRxiv. 2023 Oct 5:2023.07.20.549951. Originally published 2023 Jul 22. Preprint. [Version 2] doi: 10.1101/2023.07.20.549951 (PMC10370205; doi:10.1101/2023.07.20.549951)
Supplement: Supplementary file 1 [file NIHPP2023.07.20.549951v2-supplement-1.pdf]

bioRxiv preprint doi: https://doi.org/10.1101/2023.07.20.549951; this version posted October 5, 2023. The copyright holder for this preprint (which was not certified by peer review) is the author/funder, who has granted bioRxiv a license to display the preprint in perpetuity. It is made available under aCC-BY 4.0 International license.

Supplementary Figures and Tables

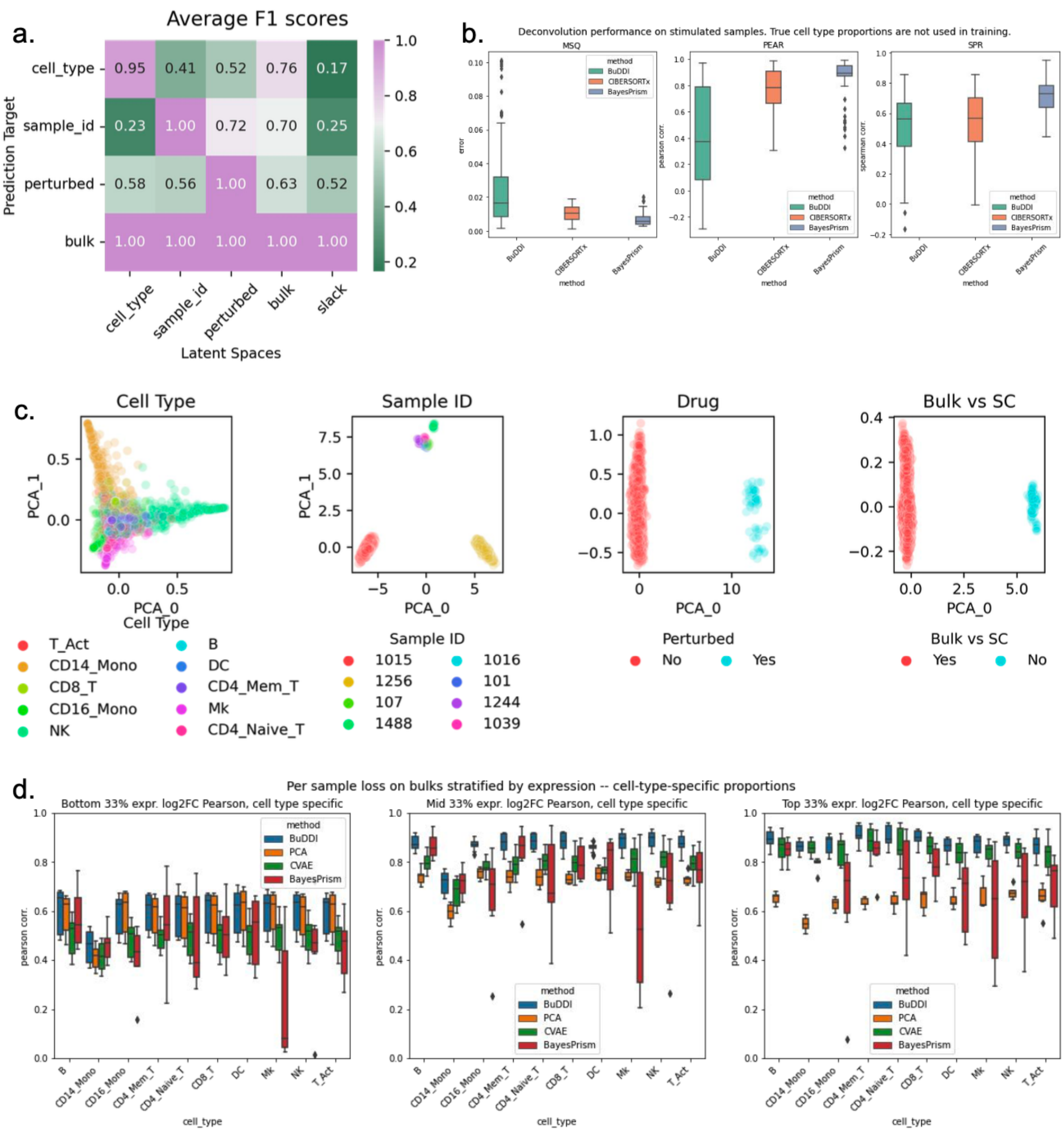

Supp Figure 1. Latent space analysis of BuDDI on Kang et al. data set with an experimental design where bulk samples are correlated with the sample IDs and perturbation status. **Panel a** depicts that average F1 score of each latent space to predict each source of variation. Midpoint coloration is the average across all observed F1 scores. Panel b compares the performance of BuDDI, CIBERSORTx, and BayesPrism, in estimating the cell type proportions. Panel c depicts each of BuDDI's latent spaces, colored by source of variation. Panel d depicts the Pearson correlation of the simulated perturbation expression, stratified by expression level.

bioRxiv preprint doi: https://doi.org/10.1101/2023.07.20.549951; this version posted October 5, 2023. The copyright holder for this preprint (which was not certified by peer review) is the author/funder, who has granted bioRxiv a license to display the preprint in perpetuity. It is made available under aCC-BY 4.0 International license.

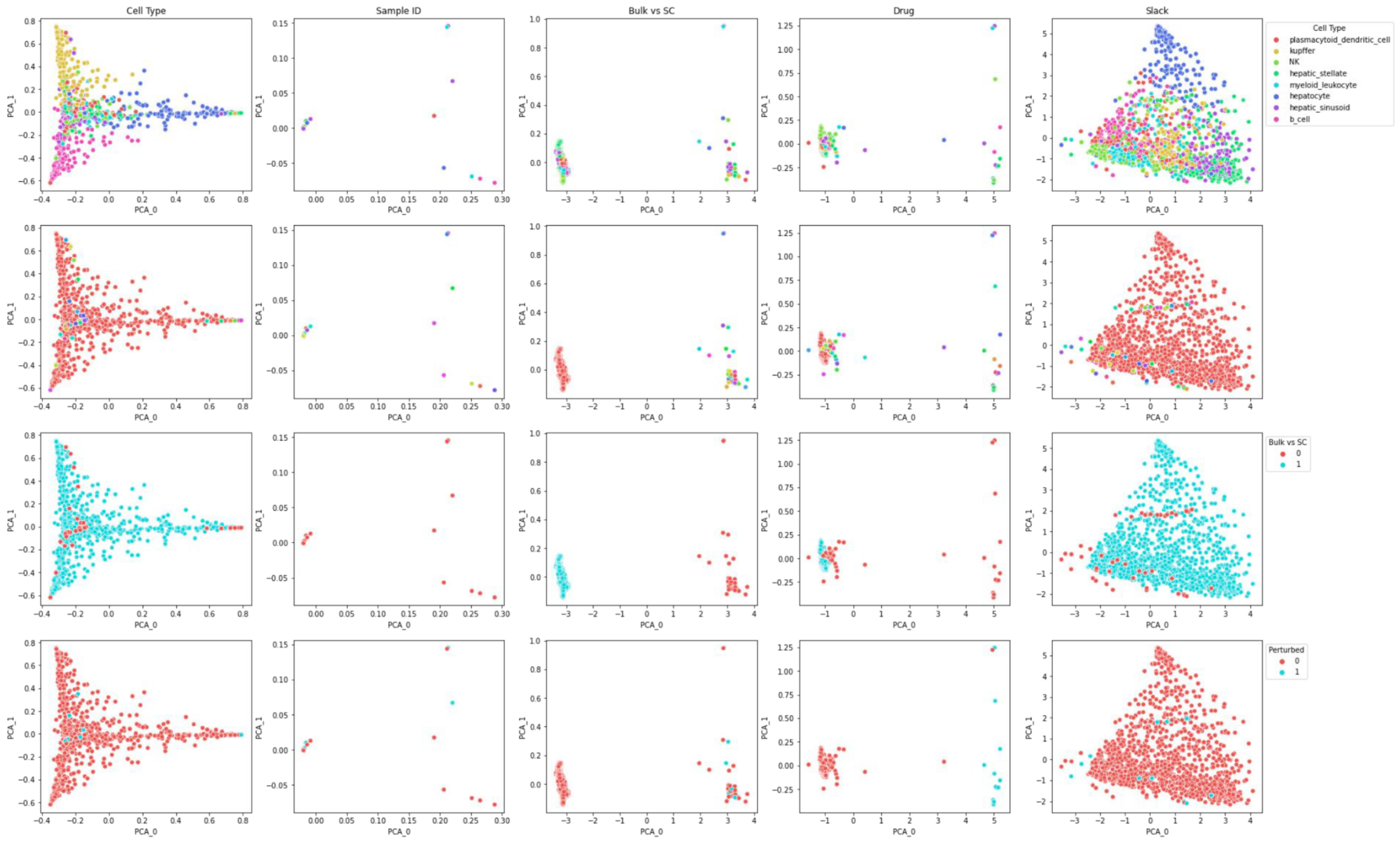

Supp Figure 2. Latent space analysis of BuDDI on Tabula Muris Senis dataset. Each column is a latent space and each row is colored by a source of variation. The second row is colored by sample ID, but due to the number of bulk samples, we omit the sample ID legend.

bioRxiv preprint doi: https://doi.org/10.1101/2023.07.20.549951; this version posted October 5, 2023. The copyright holder for this preprint (which was not certified by peer review) is the author/funder, who has granted bioRxiv a license to display the preprint in perpetuity. It is made available under aCC-BY 4.0 International license.

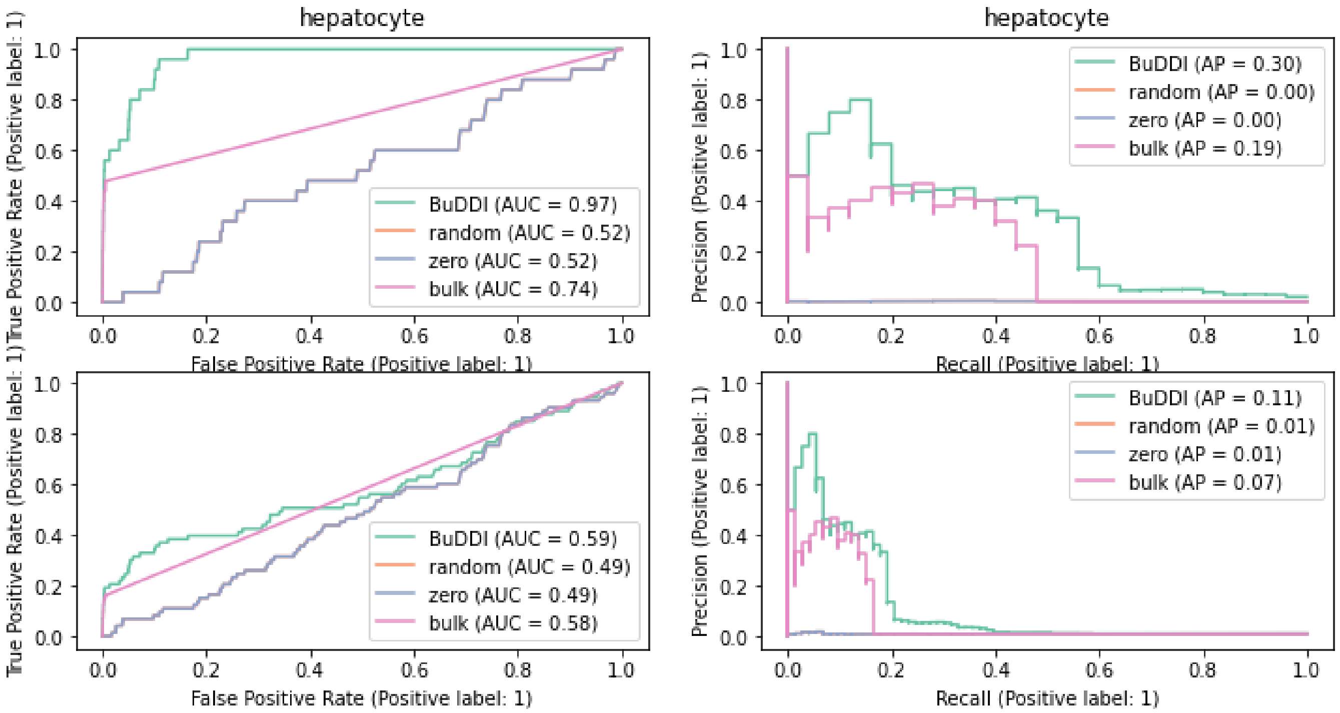

Supp Figure 3. ROC and PR curves for predicting differentially expressed genes between sexes in hepatocytes using BuDDI. Top row uses the differential expressed genes form an independent single-nucleus experiment[46] as the ground truth, bottom row uses the union of the single-nucleus and our calculated single-cell results from Tabula Muris Senis[41,42] as the ground truth.

bioRxiv preprint doi: https://doi.org/10.1101/2023.07.20.549951; this version posted October 5, 2023. The copyright holder for this preprint (which was not certified by peer review) is the author/funder, who has granted bioRxiv a license to display the preprint in perpetuity. It is made available under aCC-BY 4.0 International license.

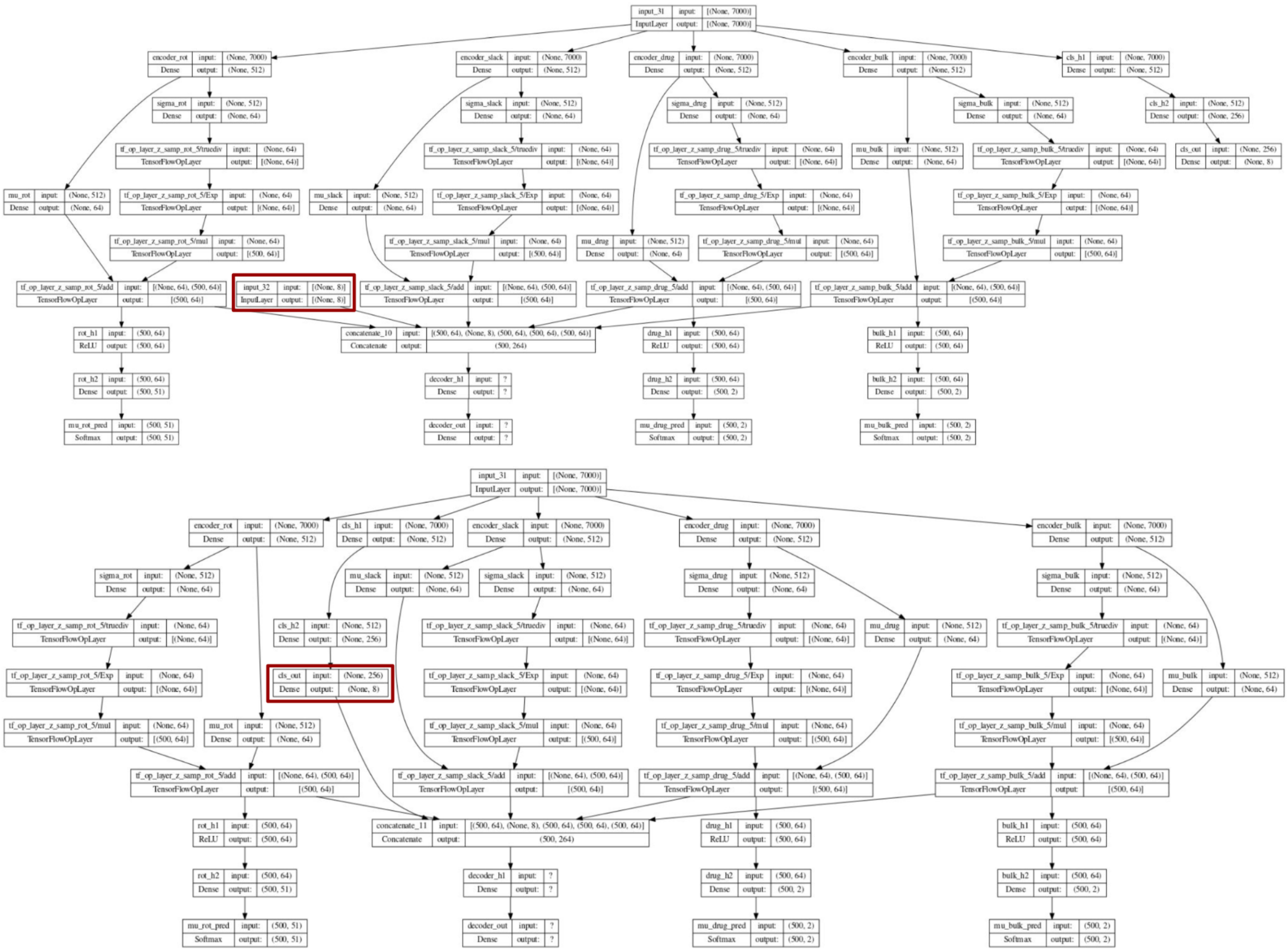

Supp Figure 4. BuDDI model overview for the supervised (top) and unsupervised (bottom) models. The red box highlights the true or estimated cell-type proportions used in BuDDI.

bioRxiv preprint doi: https://doi.org/10.1101/2023.07.20.549951; this version posted October 5, 2023. The copyright holder for this preprint (which was not certified by peer review) is the author/funder, who has granted bioRxiv a license to display the preprint in perpetuity. It is made available under aCC-BY 4.0 International license.

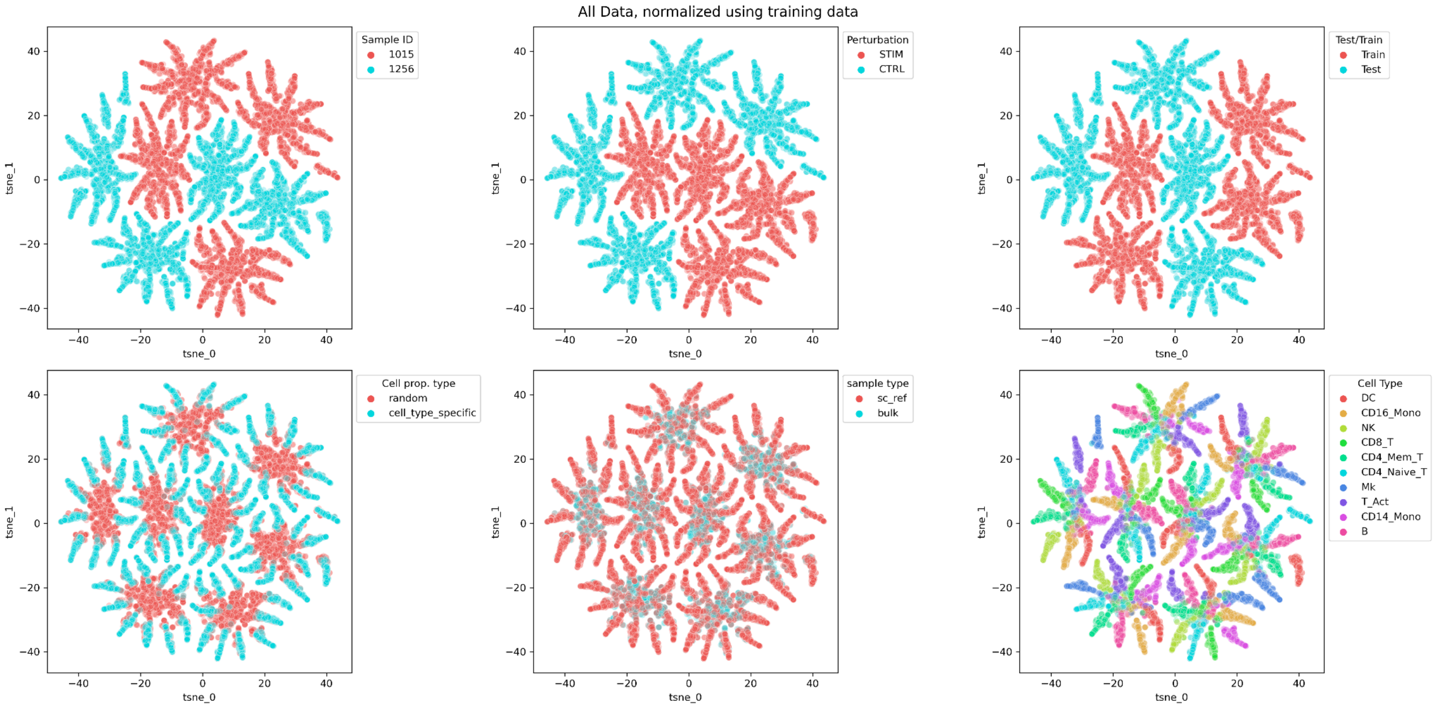

Supp Figure 5. Pseudobulk data generated and colored by source of variation. Our generated data shows independence between, each source of variation, including cell-type proportion.

bioRxiv preprint doi: https://doi.org/10.1101/2023.07.20.549951; this version posted October 5, 2023. The copyright holder for this preprint (which was not certified by peer review) is the author/funder, who has granted bioRxiv a license to display the preprint in perpetuity. It is made available under aCC-BY 4.0 International license.

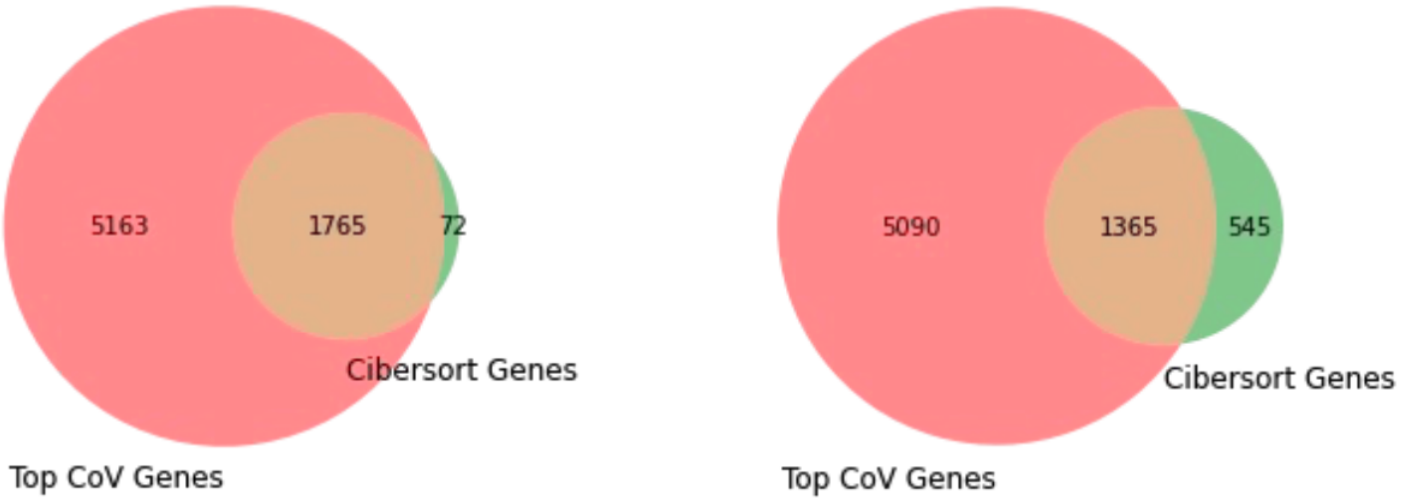

Supp Figure 6. Overlap of top coefficient of variation genes and CIBERSORTx signature genes used in the Kang et al. (left) and sex-dependent liver (right) analyses.

Cell Type in Kang et al.	Number of cells after filtering
CD14 Monocyte	4361
CD4 Naive T cells	2504
CD4 Memory T cells	1762
B cells	1363
CD16 Monocytes	1044
CD8 T cells	813
T Activated	633
NK	619
CD	604
Mk	236

Sample ID in Kang et al.	Number of cells after filtering
1015	3177
1256	2396
1499	2280
1244	2031
1016	1484
101	1224
1039	679
107	668

Supp Table 1. Number of cells by cell type and by sample ID in the Kang et al. dataset after filtering.

bioRxiv preprint doi: https://doi.org/10.1101/2023.07.20.549951; this version posted October 5, 2023. The copyright holder for this preprint (which was not certified by peer review) is the author/funder, who has granted bioRxiv a license to display the preprint in perpetuity. It is made available under aCC-BY 4.0 International license.

cell_ontology_class	B cell	Kupffer cell	NK cell	duct epithelial cell	endothelial cell of hepatic sinusoid	hepatic stellate cell	hepatocyte	myeloid leukocyte	plasmacytoid dendritic cell
mouse.id									
1-M-62	1	5	2	0	25	2	434	1	1
1-M-63	16	609	245	0	495	26	881	34	14
3-F-56	0	12	2	0	9	1	363	0	0
3-F-57	0	1	0	0	5	0	173	0	0
3-M-8/9	0	0	0	2	5	0	453	0	0
18-F-51	24	221	109	0	27	2	267	40	8
21-F-54	39	25	32	0	14	2	250	4	1
24-M-58	0	0	0	0	2	0	74	0	0
24-M-59	3	18	7	0	5	1	4	5	0
30-M-3	33	1608	56	0	3	0	1	177	7
30-M-4	14	3	11	0	5	0	0	12	1
30-M-5	73	44	88	0	80	5	29	34	9

Supp Table 2. Number of cells by sample ID and cell type after filtering and before combining the two cell types “endothelial cell of hepatic sinusoid” and “duct epithelial cell”

characteristics: sex	f	m
characteristics: age		
12	2.0	4.0
15	2.0	4.0
18	2.0	4.0
21	2.0	4.0
24	NaN	3.0
27	NaN	4.0
3	2.0	4.0
6	2.0	4.0
9	2.0	4.0

Supp Table 3. Number of bulk liver samples used in analysis by sample ID and age.
